# Surface Modifications of Anion Exchange Membranes for an Improved Reverse Electrodialysis Process Performance: A Review

**DOI:** 10.3390/membranes10080160

**Published:** 2020-07-22

**Authors:** Francis Kotoka, Ivan Merino-Garcia, Svetlozar Velizarov

**Affiliations:** Associated Laboratory for Green Chemistry-Clean Technologies and Processes (LAQV), REQUIMTE, Chemistry Department, FCT, Universidade Nova de Lisboa, Caparica 2829-516, Portugal; franciskotoka90@gmail.com (F.K.); ime.garcia@fct.unl.pt (I.M.-G.)

**Keywords:** anion exchange membranes, surface modifications, monovalent permselective membranes, antifouling behavior, improved reverse electrodialysis

## Abstract

Reverse electrodialysis (RED) technology represents a promising electro-membrane process for renewable energy harvesting from aqueous streams with different salinity. However, the performance of the key components of the system, that is, the ion exchange membranes, is limited by both the presence of multivalent ions and fouling phenomena, thus leading to a reduced generated net power density. In this context, the behavior of anion exchange membranes (AEMs) in RED systems is more severely affected, due to the undesirable interactions between their positively charged fixed groups and, mostly negatively charged, foulant materials present in natural streams. Therefore, controlling both the monovalent anion permselectivity and the membrane surface hydrophilicity is crucial. In this respect, different surface modification procedures were considered in the literature, to enhance the above-mentioned properties. This review reports and discusses the currently available approaches for surface modifications of AEMs, such as graft polymerization, dip coating, and layer-by-layer, among others, mainly focusing on preparing monovalent permselective AEMs with antifouling characteristics, but also considering hydrophilicity aspects and identifying the most promising modifying agents to be utilized. Thus, the present study aimed at providing new insights for the further design and development of selective, durable, and cost-effective modified AEMs for an enhanced RED process performance, which is indispensable for a practical implementation of this electro-membrane technology at an industrial scale.

## 1. Introduction

**Figure 1:**
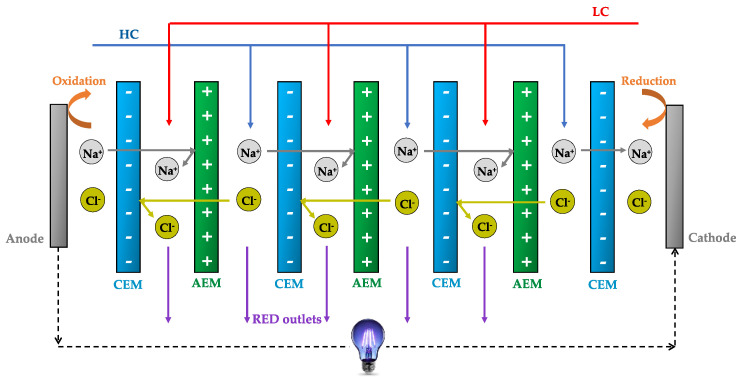
A reverse electrodialysis (RED) stack—schematic diagram (HC—high salt concentration; LC—low salt concentration).

The continuous increase of the global energy demand, which is expected to be significantly increased by 80% in 2050 [1], as well as global warming concerns, owing to the combustion of fossil fuels, is leading to the development of environment-friendly strategies and technologies, to ensure sustainable and alternative energy resources. In this context, the generation of renewable energy from aqueous natural streams with different salinity (e.g., sea and river water), using alternative series of anion exchange membranes (AEMs) and cation exchange membranes (CEMs), through reverse electrodialysis (RED), is recognized as an attractive membrane-based process [2,3,4]. Figure 1 shows the schematic illustration of a RED stack for blue energy harvesting, including the electrodes, at which redox reactions occur, thus converting the ionic current into electron current. The “CEM-AEM-CEM-AEM” arrangement is mostly preferred to prevent the permeation of the most frequently used negatively charged ferri/ferrocyanide redox couple from the electrode compartments into the high salt concentration (HC) and low salt concentration (LC) saline streams channels.

The possibility of generating power from natural streams with different salinity was first demonstrated by Pattle in 1954 [5], who obtained a power density of 0.2 W/m^2^ at 39 °C, by mixing fresh and salt water in a hydroelectric pile composed of alternate 47 CEMs and 47 AEMs, demonstrating that the pile was more likely to be economic in a warm environment, since a higher internal resistance and a lower power output was obtained at low temperatures. However, in spite of the great improvements made during the historical RED development period (graphically shown in [6]), there were still several major challenges to be tackled, concerning the technical and economic feasibility of the large-scale implementation of this technology. 

For instance, different membrane fouling phenomena, such as organic fouling, biofouling, and scaling, represent a main drawback in RED systems, because of the decrease of process efficiency and the increase of the overall cost [7]. For the first time, the fouling behavior of RED stacks that are operated for 25 days without cleaning and supplied by natural seawater and river water was investigated by Vermaas et al [8]. The performance of IEMs is adversely affected by fouling, since it leads to increases in the pressure drop and to a significant loss of the obtainable net power density, owing to an increased membrane electro-resistance, a reduced counterions permselectivity, as well as alterations in membrane properties [9,10]. In this respect, AEMs are much more sensitive to fouling issues due to the interactions between their fixed positively charged groups and the negatively charged natural organic matter (NOM) [11]. Therefore, the control, and mitigation of fouling in AEMs (including spacers and channels clogging/fouling) under real natural feedwaters, are essential to make this process feasible and preferable at industrial scale [12,13]. In this context, the two-dimensional (2D) fluorescence spectroscopy technique has shown to be effective for fouling monitoring in RED, also demonstrating that the AEM surface in contact with river water (LC stream) is significantly affected by humic compounds, in terms of fouling [14]. Additionally, the membrane fluorescence emission intensity was demonstrated to be a key parameter in the determination of the membrane cleaning efficiency [15], which is crucial to improve the long-term durability of the AEMs applied in RED stacks.

Moreover, the presence of multivalent ions in natural streams also represents a major challenge that must be overcome because the uphill transport of these ions against their concentration gradients leads to a reduced obtainable power output from RED, according to the Nernst equation [16,17,18]. Therefore, this unfavorable effect must be addressed in not only experimental studies, where a relatively high multivalent ions concentration in the LC compartments of the RED system leads to a decreased process efficiency in terms of stack voltage [19], but should also take into consideration robust modeling tools for the continuous design and optimization of the RED technology [20]. 

As a consequence, research and development of efficient strategies to render a monovalent AEM permselectivity, as well as increasing their fouling resistance is required to overcome the above-mentioned limitations. The design, development, and utilization of monovalent selective AEMs with both antifouling and mono-anion selective properties, without significantly increasing the membrane electro-resistance, represents an important challenge to move forward in achieving an improved RED process performance [21]. Additionally, comprehensive characterizations of the transport properties of AEMs are required to optimize their characteristics and to design novel membranes with beneficial properties [22]. 

The development of highly selective AEMs for RED applications is also clearly beneficial for other electro-membrane devices, such as fuel cells, especially for alkaline anion exchange membrane fuel cells (AAEMFC). These were reported to present remarkable advantages over proton exchange membrane fuel cells (PEMFC), since AAEMFC might progress the implementation of low-platinum or platinum-free fuel cell technologies, which is favorable in terms of process costs [23]. PEMFC, especially those based on Nafion-type membranes, are far studied much more, because in AAEMFC, improving the membrane chemical stability under alkaline conditions still represents the key challenge hindering the practical application of this novel fuel cell technology. In this respect, strategies followed so far for developing monovalent permselective and hydrophilic AEMs for RED applications could be easily incorporated into the AAEMFC technology, in order to move forward in seeking improvements of the AEM structure, surface properties, etc. [18].

Numerous review articles of a good standard can be found in the literature, which consider different aspects of the RED systems, like improvements of electrodes and feed solutions [2], critical operating conditions [6], current problems and challenges [24], potential of ion exchange membranes (IEMs) [7,25,26,27,28], synthesis and characterization of AEMs [29], impact of multivalent ions [18], permselectivity of IEMs [18,30,31], electro-conductive membranes [32], fouling studies [9,33], etc. Nevertheless, this paper provides an additional insight that focuses on surface AEM modification methods for RED performance improvements.

The aim of this review was therefore to classify, evaluate and discuss the different available modification approaches that could be considered to functionalize AEMs, paying special attention to the performance of the modified membranes in terms of their monovalent permselectivity, hydrophilicity and antifouling behavior, and the diverse modifying agents used till date. Additionally, the potential benefits and the associated drawbacks of the different modification techniques are also presented and discussed. The objective of the authors was to provide novel insights into the continuous design and development of innovative cost-effective, sustainable, durable, stable, and selectively modified AEMs, for an improved overall RED process efficiency. 

## 2. Membrane Surface Modification Techniques

**Figure 2:**
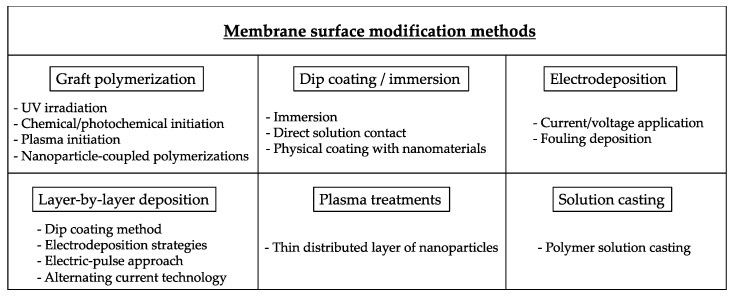
Overview of most frequently applied membrane surface modification procedures.

Surface modification of AEMs is considered to be one of the most promising approaches to induce such beneficial properties [34,35,36,37,38]. Therefore, different surface modification methods are reported in the literature to control membrane hydrophilicity concerning both permselectivity and antifouling characteristics, highlighting studies of surface polymerization through different techniques like UV-induced and oxidative self-polymerization approaches [39,40], dip coating [41], electrodeposition procedures [42], and layer-by-layer (LbL) deposition strategies [43,44,45,46], among others, as summarized in Figure 2. 

The first approach to modify IEMs with a monoselective layer was reported by Sata et al. in 1972, when the effect of applying different surface-active agents was evaluated [47]. Nonetheless, the first specifically designed IEM for RED applications was not developed and tested until the year 2012 [48], when tailor-made AEMs with controlled properties (especially membrane thickness) were prepared using an environment-friendly solution casting approach, based on amination and simultaneous cross-linking, and applied in a RED system to obtain a power density of 1.27 W/m^2^. In the subsequent years the research was mainly focused on the (i) preparation of homogeneous and thinner membranes to reduce their electrical resistance, (ii) development of alternative spacers for an enhanced obtainable power output, (iii) setting up of RED pilot plants to evaluate the technical feasibility for the practical implementation of this technology, and (iv) studies on innovative applications of RED, among others [6]. Nevertheless, additional research effort is needed to optimize the design and preparation of tailor-made AEMs. In order to achieve this goal, studies on development of either AEMs synthesis from starting materials or modification of existing AEMs are feasible; the latter approach is the focus of the present review. With the purpose of identifying the most appropriate modification procedure, besides knowledge about the properties of the unmodified membranes, a comprehensive understanding of the physicochemical properties of the modifying agents available (including their cost-effectiveness, toxicity, durability, and stability) is essential for developing highly efficient membranes for an enhanced power generation by RED.

In the following sub-sections, we highlight the different available and relevant ways for preparing modified AEMs with the desired beneficial properties of RED systems, while also identifying the strengths and weaknesses of these membrane surface modification methods, which could be classified as follows.

### 2.1. Surface Polymerization Methods

**Figure 3:**
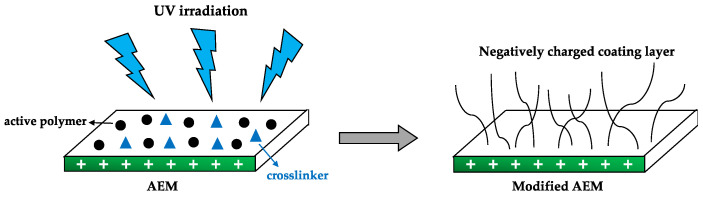
Illustrative description of a surface graft polymerization method via UV irradiation, using an active polymer (polyanion) and a crosslinking agent to create a negatively charged coating layer to the surface of an anion exchange membrane (AEM).

Surface graft polymerization induced by UV irradiation (schematically shown in Figure 3) represents an effective and useful technique to improve the properties of hydrophobic membranes, by introducing hydrophilic characteristics (thus rendering antifouling features), with the advantage of tuning the membrane surface properties with low processing costs and without damage to the bulk membrane material [39]. The possibility of improving the monovalent permselectivity of standard commercial AEMs through UV irradiation, in order to reach values close to those reported for commercial Neosepta and Selemion monovalent anion selective membranes was reported in 2014 [21]. A negative coating layer containing 2-acryloylamido-2-methylpropanesulfonic acid (AMPS) as the active polymer and *N,N*-methylenebis(acrylamide) (MBA) as the crosslinker, led to increased membrane hydrophilic properties and antifouling potential for RED applications. Additionally, decreased power density losses were observed due to the induced antifouling characteristics. Moreover, the authors suggested that the use of thinner membranes (< 110 µm) might lead to the generation of an enhanced net power density. A similar approach was considered to demonstrate that the electrochemical transport properties of AEMs were not altered after creating a thin negatively charged hydrophilic layer of urethane acrylate onto their surfaces, reporting antifouling features and highlighting the importance of controlling the UV radiation wavelength, as this parameter might affect the compactness and ion exchange capacities of the resulting modified membranes [49]. 

Different novel hydrophilic materials with antifouling properties were proposed to be grafted onto membrane surfaces, to mitigate fouling phenomena and to enhance membrane hydrophilicity, which could be used to modify AEMs for RED purposes. One of the most promising available approaches is related to the application of zwitterionic materials, which contain both negatively and positively charged groups. Thus, the zwitterionic monomers can then be polymerized via several methods like photo-initiated, plasma-initiated, UV irradiation, and graft polymerization, among others [50]. This demonstrates the possibility of reducing the membrane–water contact angle (improved hydrophilicity), and decreasing the adhesion of foulants. Additionally, from an industrial point of view, the development and use of cheaper, stable, sustainable, and eco-friendly modifying materials like polyvinyl alcohol (PVA) and chitosan was also considered to modify AEMs, with designed beneficial properties [24,51].

Overall, cross-linking-based techniques represent an attractive and efficient approach to enhance the monovalent membrane permselectivity and the membrane long-term stability, without increasing their thicknesses. The methods described in this section are commonly reported in the literature to avoid stability losses that might occur in the deposition of polyelectrolyte layer or multilayers onto membrane surfaces, due to interactions between the additional layer and the pristine membrane [52].

### 2.2. Dip Coating Strategies

**Figure 4:**
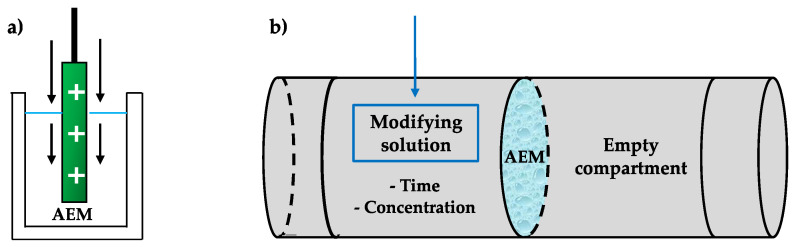
Scheme of dip coating technique highlighting the importance of controlling immersion/contact time and modifier concentration in (**a**) total immersion and (**b**) one-side modification.

Dip coating is the process of immersing a membrane during a controlled period of time into a solution containing a modifying agent, to create an additional layer with desirable characteristics onto the surface of the membrane [53,54]. In a different approach, the solution used can also be in direct contact with only one side of the membrane, using two-compartment cells, in order to prepare one-side monolayer modifications [55], as represented in Figure 4. 

The importance of optimizing the modification conditions (i.e., immersion time and modifier concentration) as well as selecting the appropriate modifying agent to obtain a stable anionic polyelectrolyte layer on the surface of different Neosepta AEMs was discussed and demonstrated in 1995 [56], when two different anionic polyelectrolytes, namely a polycondensation product of sodium naphthalene and formaldehyde, and polystyrene sulfonic acids, respectively, were considered. Regardless of the modifying agent, similar tendencies were reached, thus demonstrating an improved ion exchange capacity after modification at a concentration of 1000 ppm and an immersion time of 17 h as the optimum operating conditions. Three years later, high molecular mass anion active surfactants (selected using a low/non toxicity criteria) with alternating hydrophobic and hydrophilic groups were horizontally attached to Ionics AEMs through an immersion process, to enhance the fouling resistance of the membranes in the presence of sodium dodecylbenzene sulfonate (SDBS) as the model foulant [57]. Occurrence of fouling was suppressed for 6 h when the concentration of SDBS was 30 ppm, but after increasing the concentration up to 100 ppm, fouling was noticeable, denoting the importance of foulant concentration in natural streams used for blue energy harvesting from RED. 

More recently, this immersion technique was considered to create a negatively charged layer onto the surface of a Ralex AM-PES membrane, and Neosepta AMX. The immersion of the membrane into a polydopamine (PDA)-based solution was carried out vertically for 24 h, demonstrating an improved rejection of divalent anions, while unaffecting the monovalent anion permselectivity after modification, as theoretically expected by the authors [41], which is mandatory for an enhanced RED process efficiency. Furthermore, due to the severe problem that represents biofouling of AEMs in RED performance, the same authors studied the biofouling behavior of PDA-modified AEMs, prepared by following the same immersion procedure. *Pseudomonas putida* was utilized as a model biofoulant to evaluate the bacterial coverage percentage via scanning electron microscope (SEM) technique, during RED stack operation, where the possibility of reducing the bacterial attachment was demonstrated [58].

One-side modification approaches based on this technique were also recently proposed to create a negatively charged thin monolayer on the surface of AEMs. In particular, heterogeneous Ralex AEMs were modified by direct contact (during 24 h) with poly(acrylic) acid (PAA)-based solutions, which represents the use of a non-toxic and stable modifying agent [59,60]. The authors reported an improved surface hydrophilicity and monovalent membrane permselectivity as a function of different concentrations of PAA, without compromising the membrane electro-resistance [55]. 

In short, dip coating strategies represent an easy, fast, and effective method to incorporate a charged layer onto the surface of AEMs, leading to improved characteristics like higher ion-exchange capacities, antifouling and antibiofouling properties, and rejection of divalent anions. However, since there is a trade-off between the stability of the added layer and the monovalent anion permselectivity, a careful control of the modification conditions (i.e., immersion time, modifying agent concentration, pH, and temperature) is required for the design of novel and effective modified AEMs via the dip coating method. 

In this context, dip coating or immersion of membranes into the modifying solutions can also be carried out to incorporate several layers onto the membrane surfaces, in order to further improve the behavior of the modified AEMs. This alternative process is known as the layer-by-layer (LbL) deposition method, which is discussed in the following section of this review.

### 2.3. Layer-by-Layer (LbL) Approaches

**Figure 5:**
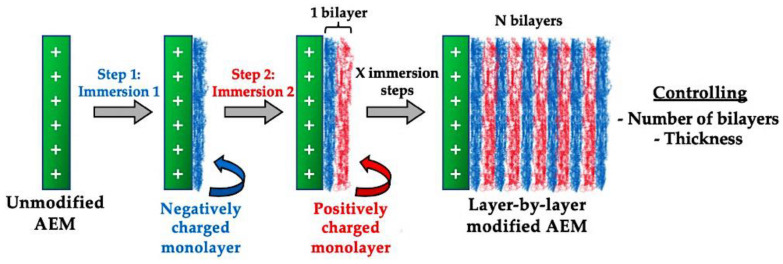
Graphical representation of a layer-by-layer (LbL) strategy via vertical membrane immersion, in which X stands for the number of consecutive immersion steps.

The preparation of innovative modified AEMs with enhanced monovalent permselective or antifouling properties, through LbL-based approaches, represents a versatile and efficient way for improving RED performance, since it allows for tuning of the membrane properties, such as swelling, thickness or surface charge density, among others [43,44]. The number of layers added to the membrane surface was shown to be an important parameter that must be optimized in LbL modification strategies, as graphically shown in Figure 5. In this respect, alternative and repeated layers of poly(sodium 4-styrene sulfonate), PSS as a polyanion, and poly(allynamine hydrochloride), (PAH) as a polycation were deposited onto a standard AEM (Neosepta AMX) membrane. The results showed an improved constant monovalent anion permselectivity after 15 layers, using an LbL deposition approach (PSS as the top layer to obtain a negatively charged surface) [61], which the authors associated with an increase in the total excess areal negative surface charge. These 15-layer-based AEMs also showed antifouling characteristics; however, the optimal conditions for antifouling potential were reached with seven layers, in which the lowest membrane–water contact angle was achieved. 

Similar findings were observed when modifying a CJMA-2 standard AEM with thin-surface multilayers, based on negatively charged PSS and positively charged poly(ethyleneimine) (PEI) [37]. The modified membranes with 7.5 bilayers showed a comparable monovalent anion permselectivity to the one associated with commercial ACS monovalent selective membranes, and considerably enhanced the anti-organic fouling resistance (improved by around 30%) at the same time. Due to the favorable characteristics of the prepared membrane, RED tests were carried out using NaCl and Na_2_SO_4_ aqueous solutions, in the presence of humic acid as a model foulant, highlighting an improved net power density up to 17% and an enhanced energy conversion efficiency after LbL modification. The authors suggested that the RED process efficiency could be further improved by modifying the membrane matrix instead of its surface, with the purpose of preparing home-made thinner modified AEMs, with a lower membrane electro-resistance. 

Moreover, an alternative and innovative electric-pulse LbL approach was proposed in 2018 to enhance the stability of a multilayer, using chitosan-based biopolymers (i.e., N-O-sulfonic acid benzyl chitosan (NSBC) and hydroxypropyl trimethyl ammonium chloride chitosan (HACC)) coated onto one side of a commercial AEM [45]. This electric-pulse (provided by an electrochemical workstation) deposition technique allowed the activation of both NSBC and HACC, showing a tremendous adsorption capacity. When considering 7.5 bilayers, a homogeneous and stable multilayer with a relatively low surface electrical resistance was obtained. One year later, an alternating current LbL (AC-LbL) technology was first proposed to prepare a monoselective AEM with antifouling properties in long-term use and AC frequency of 50 Hz, at which the polyelectrolytes were assembled as high as 50 times per second [44]. In this study, 7.5 bilayers of the hydrophilic poly(4-styrenesulfonic acid-co-maleic acid) sodium salt and 2-hydroxypropyltrimethyl ammonium chloride chitosan were homogeneously deposited onto the surface of the membrane and then crosslinked using 1,4-bis(2′,3′-epoxypropyl) perfluoro-1-butane, demonstrating an antifouling ability, and a stable operation for 96 h. 

To sum up, the strengths of different LbL-based methods for AEM modification with RED improvement purposes are—(i) an improved monovalent permselectivity might be achieved, which is directly related to a higher divalent ions rejection; (ii) the membrane hydrophilicity can be controlled in order to develop modified AEMs with desired organic antifouling characteristics; (iii) homogeneous and stable thin multilayers can be assembled onto the membrane surface. However, the selection of the modifying agent/s and the operating modification conditions is not effortless. Research on utilization of cheaper, non-toxic, sustainable and environment-friendly polymers is still required to design tailor-made AEMs using greener preparation methods. Last but not the least, the electro-membrane resistance must be controlled, owing to the high number of layers often involved in LbL strategies, in which 7.5 bilayers seems to be the optimal value, in order to keep the monovalent membrane permselectivity unaffected [18].

### 2.4. Electrodeposition Procedures

A simple electrodeposition method, which employs an electrical field to help deposit a layer on an anion-exchange membrane surface was proposed [53]. In particular, PEI was covalently electrodeposited on the surface of a home-made AEM based on partially quaternized poly(phenylene oxide) (QPPO), to favor the migration of monovalent anions across the membrane, while hindering the transport of multivalent ones [35]. The monovalent permselectivity (Cl^−^/SO_4_^2−^ system) was increased from 0.79 to 4.27 after modification, due to a decreased sulfate-ion leakage rate (from 39.6% to 19.4%). The long-term stability of the monovalent selective modified AEMs was demonstrated for 70 h of continuous operation, in terms of Cl^−^ and SO_4_^2−^ concentration evolution in the dilute chamber, thus, evidencing that this PEI electrodeposition strategy was not only effective and feasible for the preparation of monovalent selective AEMs, but also presents an appealing potential in the long-term operation. 

Heterogeneous AEMs were modified in a six compartmental apparatus via electrodeposition with graphene oxide (GO) and a supporting NaCl electrolyte [62]. It was found out that low concentrations of NaCl (0.01 M) and higher GO concentrations (0.1–0.5 g/L) could enhance the modification effect in terms of hydrophilicity and negative charge density, as higher NaCl concentrations would favor the competitive migration of Cl^−^ ions. Additionally, increasing the current density (1–5 mA/cm^2^) resulted in enhanced properties of the modified membranes. The authors suggest that further increases of this parameter would lead to an uneven distribution of the GO layer. As more hydrophilic surfaces with negative zeta potential were prepared, fouling resistance was also improved in the presence of 150 mg/L of SDBS as a model foulant, which denoted the feasibility of these GO-modified AEMs to be used in RED configurations. On the other hand, the same research group also studied the feasibility of combining two modification methods (electrodeposition and coating) to incorporate a thin negatively charged and hydrophilic PDA layer onto the GO-AEMs (modified by electrodeposition), with the purpose of improving their antifouling properties and reducing the roughness of the modified membranes [63]. The authors demonstrated an extensive enhancement of the membrane stability with antifouling behavior in an electrodialysis apparatus composed of one AEM and two CEMs, which operates at a constant voltage of 4 V for 20 h when a smoother and denser PDA/GO layer was incorporated, compared to the GO-modified membrane, without taking into consideration the inclusion of PDA. Furthermore, the possibility of increasing the performance of AEMs after membrane modification by electrodeposition using a polyelectrolyte containing different functional groups like PSS and poly(sodium acrylate) (PAAS) was also clearly demonstrated in electrodialysis [64], as their higher negative charge surface density led to an enhanced membrane antifouling behavior. 

**Figure 6:**
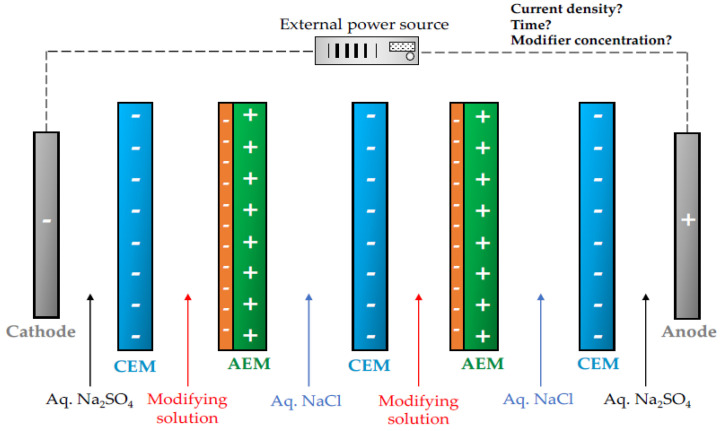
An example of a membrane stack, in which an external potential difference is applied for creating a negatively charged modifying layer on AEMs by electrodeposition.

In brief, the modification conditions like the current/voltage applied to carry out the modifier electrodeposition, as well as the amount of the deposited agent and its composition, which determined the thickness of the modified AEM, must be carefully monitored and optimized to develop stable negatively charged layers with hydrophilic, monovalent anion permselective and antifouling properties, via electrodeposition. Furthermore, since the electrodes play an important role in this modification approach (the anode attracts the modifying agent according to its opposite charge, as shown in Figure 6), more comprehensive studies about electrode durability and stability are required to improve the efficiency of the electrodeposition procedures. 

### 2.5. Alternative Modification Techniques

The previously outlined modification procedures showed the possibility of tuning different properties of AEMs like hydrophilicity, monovalent permselectivity, surface charge density, electrical conductivity, fouling resistance, etc. Nevertheless, the long-term stability and durability of the modified membranes is usually limited by undesirable interactions between the modifying layers and the membrane surface. For instance, since an incorporated adsorbed layer on the surface of an AEM could be desorbed during the RED operation, a chemically modified surface, as a result, seemed to be favorable [53]. Additionally, an increase in membrane area resistance often led to a reduction of the ion-exchange capacity [65]. Therefore, several alternative modification methods (presented in the following paragraphs) were proposed to provide novel insights into the membrane modification field, which could be utilized for improvements in membranes designed for RED applications.

Although fouling generally represents an undesirable phenomenon in the electro-membrane field, an innovative approach called “fouling deposition” was recently proposed and investigated to convert the adverse perspective associated with fouling into an useful tool, to prepare permselective membranes [65]. In this study, Neosepta AMX AEMs were fouled with sulfonated poly(2,6-di-methyl-1,4-phenylene oxide) (SPPO) at different constant current density levels (10, 30, and 50 mA/cm^2^), using an electrodialysis stack with increased spacer thickness and decreased inlet flow velocity, in which the effect of current density on the permselectivity (Cl^−^/SO_4_^2−^) was also investigated at 5, 10, and 20 mA/cm^2^. The best membrane behavior was achieved at 10 mA/cm^2^ (fouling deposition and permselectivity study conditions), at which a high permselectivity coefficient (Cl^−^/SO_4_^2−^) value of 52.44 was reached, which was superior not only to that of an unmodified Neosepta AMX membrane but also when compared to a commercial monovalent anion permselective Neosepta ACS membrane. The authors claimed that a better understanding of the fouling mechanisms and the role of the fouling layer on membrane permselectivity was still required to move forward in this fouling deposition technology.

The use of nanomaterials, such as nanoparticles (NPs), was reported to be one of the most promising techniques to modify membrane properties, like permselectivity, roughness, and morphology, including antifouling characteristics, even though the NP loading must be controlled to avoid inaccessibility to fixed functional groups, which might lead to membrane conductivity and permselectivity losses [66]. For example, a commercial polyethylene AEM was modified by physical coating using sulfonated poly(2,6-dimethyl-1,4-phenylene oxide) (sPPO) and two nanomaterials of different geometry, with optimized loadings (oxidized multi-walled carbon nanotubes, CNTs–COO^−^, or sulfonated iron oxide NPs, Fe_2_O_3_–SO_4_^2−^), showing alterations/improvements in the membrane surface after modification (negatively charged) in terms of hydrophilicity and homogeneity, without compromising the membrane electro-resistance. This led to a relevant improvement of fouling resistance to sodium dodecyl sulfate (SDS) by more than 45% [67]. Due to the fact that similar performances were obtained with both nanomaterials, the authors highlighted the need to perform economical evaluations with the purpose of selecting the most appropriate material. 

Plasma treatments also offer the possibility to produce a thin uniformly distributed layer of NPs on the membrane surface, using a vacuum reactor, a surface modifier, and gas plasma, in which the thickness of the created layer was affected by the deposition rate and time [53]. Other proposed possibilities were represented either by incorporating NPs into the polymerization procedures with heating, or through sol-gel reactions [66].

Finally, solution casting methods could also be utilized to modify the surface of a membrane by depositing a layer with controlled thickness and structural properties, following the same procedure as that for preparing home-made membranes through the phase-inversion approach [53]. Although most cases are focused on modifying CEMs with a chitosan-based layer in which the thickness of the top layer could be controlled by varying the chitosan concentration [68], this approach could also be adapted to design and functionalize AEMs. For instance, an environment-friendly and safe solution casting approach was developed in 2012 to prepare innovative AEMs, which represented the first reported attempt showing the performance of tailor-made AEMs in a RED stack [48]. The casting solution was based on polyepichlorohydrin (PECH) as the active polymer, polyacrylonitrile (PAN) as the inert polymer, and a tertiary diamine with the function of introducing the ion-exchange groups by amination, as well as carrying out the cross-linking. The importance of controlling the excess of tertiary diamine added to the casting solution, as well as the membrane thickness, were clearly demonstrated with the aim of obtaining AEMs with lower thicknesses and membrane electro-resistances, and higher permselectivities. The application of these membranes in RED led to a power density as high as 1.27 W/m^2^, which was higher than the power output obtained when using a commercial Neosepta AMX membrane.

More recently, a novel, environment-friendly and cost-competitive casting approach was designed to functionalize AEMs through organic–organic hybridization, using low-cost and non-toxic polymers like poly(diallyldimethylammonium chloride) (PDDA) and PVA, which were blended in different mass ratios, to create a novel AEM for a lab-scale RED stack [69]. The importance of controlling the mass ratio of the two polymers was clearly demonstrated, because a higher PDDA loading led to a lower membrane electro-resistance and a higher IEC, reaching a gross power density of 0.58 W/m^2^ at the optimum conditions (PDDA/PVA ratio of 1.5). This represented a higher value than the 0.40 W/m^2^ achieved with a commercial Fumasep FAS membrane (used as a reference) in the same RED configuration, thus highlighting the potential of designing hybrid AEMs for RED applications. 

## 3. Selected Studies on Modified AEMs with Improved Performance

A number of different AEMs were selected so far for surface modifications, in order to improve their behavior in terms of monovalent permselectivity, membrane electro-resistance, hydrophilicity, antifouling characteristics, and so on. Table 1 summarizes several attractive AEM modification studies, paying special attention to the membrane electro-resistance results before/after modification, since controlling the trade-off between this parameter and the monovalent permselectivity is crucial for achieving an improved RED process performance. Thus, the monovalent membrane permselectivity as well as the special improvements reached in each case are also shown. Although most research is focused on electrodialysis (ED) applications, the different membrane modification strategies could be easily adapted towards RED perspectives.

The following important insights can be gained from Table 1 regarding the development of different modified AEMs, which could be applied in ED and RED stacks:

- Polymerization-based modification methods are capable of considerably improving the membrane behavior in terms of multivalent ions rejection (e.g., SO_4_^2−^), i.e., the membrane permselectivity (Cl^−^/SO_4_^2−^) is clearly enhanced after modification. However, an unfavorable impact in the membrane electro-resistance is often observed, which might be associated with an increased thickness of the modified AEMs compared to the pristine one. The effect of the modifying agent selected is clearly shown in Table 1. For example, the modification of a standard-grade homogeneous Fuji A membrane with AMPS and MBA via UV-curing with specific RED performance improvement purposes, resulted in an increased permselectivity (a comparable value with the one associated with a commercial Neosepta ACS membrane was reached), including enhanced hydrophilicity and antifouling characteristics, almost without compromising the membrane electro-resistance [21].
- Several AEMs were also proposed to be modified via immersion/dip coating-based strategies, with the purpose of enhancing their surface hydrophilicity, antifouling behavior, and rejection of divalent anions. Nevertheless, more comprehensive studies on membrane electro-resistances (preferably via electrochemical impedance spectroscopy) are required after modification to focus on developing AEMs with a lower electrical resistance for RED, which might lead to an increased obtainable net power density.

**Figure 7:**
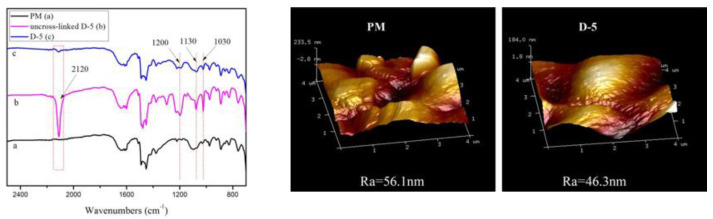
FTIR spectra (**left**) and AFM images (**right**) of a commercial AEM (PM), before and after modification. Reproduced with permission from [70].

In order to support the positive effects of modifying AEMs through the two methods above-mentioned, Figure 7 shows the Fourier-transform infrared spectroscopy (FTIR) spectra and the atomic force microscope (AFM) images of a commercial AEM (JMA-II-07), which was modified by infiltration (immersion), using 4,4-diazostilbene-2,2-disulfonic acid disodium salt (DAS) as the modifying agent, and UV-cross linking [70]. The FTIR revealed that both the infiltrated (uncross-linked D-5), and UV-crosslinked (D-5) membranes exhibited sulfonate absorption bands at 1200, 1130 and 1030 cm^−1^. However, these bands were not observed in the pristine membrane (PM). Additionally, the uncross-linked D-5 membrane showed a characteristic peak of azido groups in the DAS at 2120 cm^−1^, thus demonstrating the presence of the modifying agent on the membrane surface. By contrast, this peak was not clear in the D-5 FTIR spectrum, implying that the azido groups might have reacted with the membrane surface, forming stable covalent bonds under UV irradiation.

On the other hand, the AFM results showed the surface homogeneity and roughness of the PM and D-5 membranes. Thus, the arithmetic mean roughness (Ra) of the AEM was reduced from 56.1 nm to 46.3 nm (17.5% reduction) after modification. The authors suggested that the reduction in roughness indicated that the DAS modifying agent rendered higher uniformity, increased tightness, and smoothness on the membrane surface. Consequently, the modified UV cross-linked membrane (D-5) reached an increased $P_{\mathrm{SO}4^{2-}}^{{\mathrm{Cl}}^{-}}$ (11.21), compared to the performance of the PM (0.55), thus, denoting the effectiveness of the modification considered.

**Figure 8:**
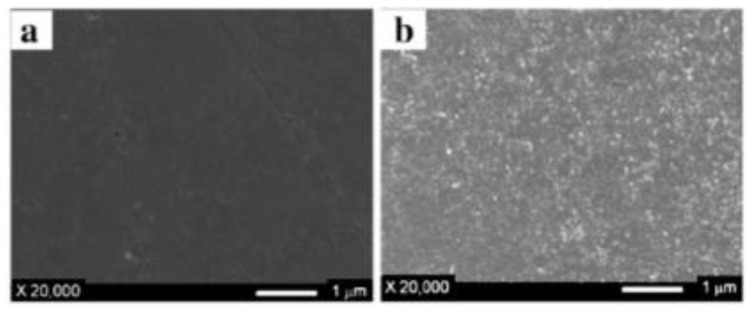
SEM images of (**a**) unmodified membrane, and (**b**) PDA (0.1 kg/m^3^) modified AEM. Reproduced with permission from [73].

Furthermore, the scanning electron microscope (SEM) micrographs of a Neosepta AMX surface, before and after modification by immersion, using polydopamine (PDA) as the modifying agent is shown in Figure 8. The authors reported that the thickness and roughness of the modified layer is increased at higher PDA concentrations [73]. However, this fact did not compromise the antifouling potential of the modified membranes (optimum PDA concentration was found to be 0.1 kg/m^3^). In this context, the antifouling potential transition time was improved from less than 25 min to about 300 min, employing SDBS as the model foulant.

In the field of layer-by-layer (LbL) techniques developed so far, the main key challenge is related to the need of applying greener polymers, especially in the formation of positively charged layers. Although the addition of successive layers onto the surface of a membrane leads to an increase in the thickness of the prepared membrane, this parameter can be controlled in this method by taking into account the modifying agent concentration, its deposition time, and the number of deposited layers, among others. Surprisingly, in some cases, the membrane electro-resistance is not significantly affected after modification.

**Figure 9:**
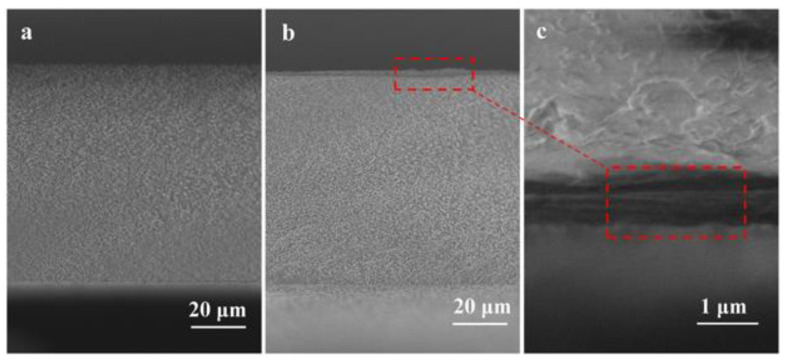
SEM cross-sections of: (**a**) original CJMA-2 AEM, (**b**) modified CJMA-2-7.5 AEM, and (**c**) high magnification of the modified membrane. Reproduced with permission from [37].

For example, the SEM images of cross-sections of a standard AEM before (CJMA-2) and after modification (CJMA-2-7.5) by LbL deposition (7.5 bilayers) are represented in Figure 9. The modification method was carried out by using poly(styrenesulfonate) (PSS) and poly(ethyleneimine) (PEI) as the modifying agents [37]. The detectable thickness of the modified layer ranged from 0.88 μm to 1.20 μm. After surface modification, the antifouling potential was increased by 38% using sodium dodecylbenzene sulfonate (SDBS) as the model foulant, without significantly compromising the membrane electro-resistance (from 2.8 to 3.3 Ω·cm^2^), whereas the gross power density was enhanced by 10%, even in the presence of humic acid (HA) as the model foulant.

The electrodeposition technique, so far carried out for ED systems, is usually useful to render anti-organic fouling properties of the membranes and to improve their long-term operation stability by controlling the applied current/voltage and time. In this regard, an improved permselectivity, as well as an enhanced stability with antifouling behavior results were shown in the literature, even though higher membrane electro-resistances were reported. For instance, a Neosepta AEM, modified with poly(ethyleneimine) via electrodeposition showed an improved permselectivity between Cl^−^ and SO_4_^2−^ (from 0.79 to 4.2). The modified layer presented a high stability after 70 h of operation, with an increase in the membrane electro-resistance from 4.63 to 6.05 Ω·cm^2^.

**Figure 10:**
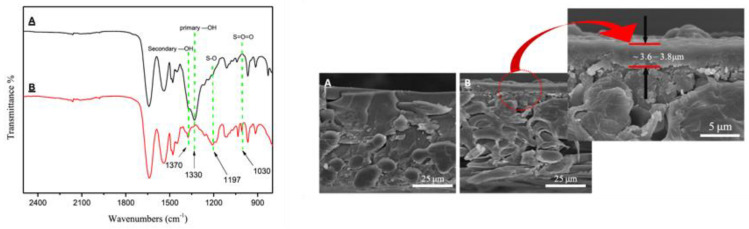
FTIR spectra (**left**) of the homogeneous unmodified AEM (A) and modified AEM (B). Cross-sectional SEM micrographs (**right**) of the unmodified (A) and modified (B) AEM. Reproduced with permission from [42].

The improved properties of modified AEMs (by electrodeposition) were also demonstrated through FTIR and SEM analyses. For instance, Figure 10 represents the FTIR spectra and SEM micrographs of unmodified (A), and modified (B) Fujifilm AEMs via an alternate electrodeposition approach, using poly(sodium 4-styrene sulfonate) (PSS) and hydroxypropyltrimethyl ammonium chloride chitosan (HACC) as the modifying agents [42]. The FTIR spectrum of the modified membrane (red profile) showed strong sulfate absorptions at 1197 and 1030 cm^−1^, thus, demonstrating the presence of S=O and SO_3_H in the membrane surface. Additionally, a primary –OH band was observed at 1330 cm^−1^ for the unmodified AEM, whereas a secondary –OH bending vibration was observed at 1370 cm^−1^ for the modified membrane, which the authors associated with the presence of HACC on the membrane surface, thus, demonstrating the successful electrodeposition. This fact was further demonstrated by SEM images (Figure 10-right), in which the PSS/HACC incorporated multilayer (3.6–3.8 μm) could be clearly identified in the surface of the modified membrane (image B), in comparison with its absence in the pristine membrane (image A). As a result, $P_{\mathrm{SO}4^{2-}}^{{\mathrm{Cl}}^{-}}$ was enhanced from 0.66 to 2.90, after membrane surface modification.

A novel approach called “fouling deposition” was proposed in 2019 as an effective novel modification technique, to prepare monovalent permselective membranes [65]. In this respect, the permselectivity between Cl^−^ and SO_4_^2−^ of a Neosepta AMX AEM was greatly improved from 1.95 to 52.44. In addition, the membrane resistance was not significantly compromised (from 2.40 to 2.83 Ω·cm^2^), thus, denoting the potential of this novel approach to move forward in the preparation of AEMs with desired properties for RED applications.

Furthermore, the analysis of the antifouling potential of the modified AEMs is essential to develop membranes with antifouling characteristics and beneficial properties. Although this parameter depends on several factors [54,58,71], in most studies reported in Table 1, the membrane–water contact angle was decreased (improved hydrophilicity) after membrane modification. Therefore, this parameter represents a key figure of merit to evaluate the antifouling membrane potential. In general, a decreased membrane–water contact angle (increased membrane hydrophilicity) leads to a higher organic antifouling resistance of the modified membrane. The antifouling potential was observed to be significantly improved after modifications, resulting in a contact angle value in the range of 20–70 θ [21,37,40,67,71,74]. Overall, further systematic dedicated studies are required to understand the relation between the membrane hydrophilicity and the antifouling potential of different AEMs, in greater details.

## 4. Future Outlook and Perspectives

A number of interesting strategies has been so far proposed to improve the performance of different AEMs, as summarized and discussed in this review. Several remaining key challenges requiring further research were identified and need to be addressed, in order to design and commercialize permselective, sustainable, and cost-effective AEMs, for the practical implementation of the RED technology at a large-scale level, namely:

(i) A deeper understanding of the interactions between the modifying agents and the membrane materials is clearly required, in order to elucidate in detail the fouling mechanisms and the behavior of foulants under real (with natural feedwaters) operating conditions, with the aim of developing AEMs with antifouling characteristics.

(ii) The development of greener modifying agents still represents a major challenge that should be overcome with the purpose of preparing cheaper, non-toxic, and durable AEMs. For example, the layer-by-layer approach discussed in this work consider the subsequent addition of negatively and positively charged layers on a membrane surface. In this respect, toxic materials are usually involved, especially in the formation of positively charged layers. Therefore, the currently available modification procedures should be adapted towards the application of environment-friendly modifying materials, taking into account the effects of the operating conditions. 

(iii) Efficient pre-treatment and appropriate membrane cleaning methods must be adopted or developed to improve the performance of AEMs, as well as their long-term durability and stability, taking into consideration Life Cycle Assessment and economic perspectives, in terms of investment and operating costs.

(iv) The trade-off between membrane permselectivity and membrane electro-resistance represents an important aspect that must be considered to enhance the obtainable net power output from RED. In this context, further comprehensive transport and electrochemical (e.g., electrochemical impedance spectroscopy) studies should be addressed. In this regard, advanced modeling tools should be explored to simulate and quantify the ohmic and non-ohmic resistances involved in the process.

Therefore, the following promising perspectives/lines for future research could be proposed to achieve an improved RED process performance:

(i) Design and preparation of eco-friendly home-made AEMs, not only focusing on surface modification, but also exploring the possibility of modifying the interior of the polymeric membrane matrix. Further surface modification steps could also be considered after the matrix preparation/modification.

(ii) Study and development of non-toxic hydrophilic materials with antifouling properties to be coated or grafted onto the surface of AEMs, including the evaluation of the mechanisms of their antifouling behavior and the assessment of the material stability in the long-term.

(iii) Combination of modification approaches to improve the performance and behavior of AEMs in terms of permselectivity, electro-resistance, hydrophilicity, and antifouling characteristics, including the addition of nanomaterials.

(iv) Evaluation of the performance of RED stacks with significant number of cell pairs, using natural seawaters and river waters or relevant industrial saline brines, in order to validate not only the stability and durability of the prepared membranes, but also the efficacy of their periodic cleaning, in order to allow for their prolonged re-use.

(v) Dedicated studies on energy recovery by using the proposed modified AEMs in RED stacks are required to evaluate the technical and the economic feasibility of the process under real conditions. Although investigations on membrane level are essential to evaluate membrane properties before and after modification, the quantification of the “energy savings” must be addressed to move forward towards making the implementation of the RED technology preferable at an industrial scale.

## Figures and Tables

**Table 1:** Modified AEMs with improved monovalent permselective, hydrophilic, or antifouling properties with promising potential to be used in RED systems.

Membrane	Modification Method/ Modifying Agent	Permselectivity $\left(P_{SO4^{2-}}^{Cl^{-}}\right)$ or Other Figures of Merit	Special Improvement/s	Membrane Electro-Resistance Change (Ω·cm^2^)	Reference
JMA-II-07 (Tingrun Co. Ltd. Beijing, China)	Infiltration and cross-linking under UV irradiation/ 4,4-diazostilbene-2,2-disulfonic acid disodium salt (DAS)	From 0.55 to 11.21 (better behavior than Selemion^®^ ASV)	The modified layer was stable after 80 h of operation.	From 3.53 to 4.50	[70]
Polyvinyl alcohol and quaternized-chitosan based AEM	Electronegative coating through interfacial polymerization/ 3,5-diaminobenzoic acid (DMA)	From 1.80 to ~9.30	Improved antifouling potential transition time (from 55 min to 92 min), enhanced hydrophilicity (contact angle decreased from 56 θ to 40 θ) and high thermal/mechanical membrane stability.	From 1.88 to 4.29	[71]
Polyvinyl alcohol and quaternized-chitosan based AEM	Electronegative coating through interfacial polymerization/ 2,5 diaminobenzenesulfonic acid (DSA)	From 1.80 to 10.30	Improved antifouling potential transition time (from 55 min to 95 min), enhanced hydrophilicity (contact angle decreased from 56 θ to 38 θ) and high thermal/mechanical membrane stability.	From 1.88 to 3.21	[71]
AEM Type I (Fujifilm Corp.)	Rapid deposition and polymerization/ L-polydopamine (L-PDA), and 4-amino-benzenesulfonic acid monosodium salt (ABS)	From 1.00 to 4.66	Improved organic antifouling potential (electrical resistance due to fouling decreased from 4.78 Ω·cm^2^ to 0.53 Ω·cm^2^), enhanced hydrophilicity (contact angle decreased from 105.2 θ to 68.6 θ). Separation efficiency enhanced from 2% to 63%.	N.A.	[40]
Fuji A (Fujifilm Corp.)	Coating by UV-curing/ 2-acryloylamido-2-methylpropane sulfonic acid (AMPS) and N,N-methylenebis(acrylamide) (MBA)	$P_{{\mathrm{Cl}}^{-}}^{\mathrm{SO}4^{2-}}$ decreased by 10% and was comparable to that of Neosepta ACS	Improved organic antifouling potential transition time from 50 min to 90 min, increased hydrophilicity (contact angle reduced from 63 θ to 24 θ).	From 0.93 to 1.10	[21] *
Heterogeneous Ralex AM-PP (Mega a.s.)	Physical coating/ sPPO, sulfonated -Fe_2_O_3_ and oxidized carbon nanotubes (CNTs)	N.A.	Antifouling resistance improved by 53%. Enhanced hydrophilicity properties (contact angle decreased from 100.1 θ to 57.9 θ) with 40–60% energy savings were achieved.	N.A.	[67]
AEM Type I (Fujifilm Corp.)	Self-adhesion deposition/ Sulfonated polydopamine (SPDA)	From 1.00 to 34.02 (improving both Neosepta ACS and Selemion ASV performances)	Higher anti-organic fouling potential (transition time improved from 76 min to 112 min).	From 1.02 to 6.83	[72]
AEM Type I (Fujifilm Corp.)	Self-adhesion deposition/ Polydopamine (PDA)	From 1.00 to 11.59 (better results than Neosepta ACS and Selemion ASV)	Higher anti-organic fouling potential (transition time improved from 76 min to 106 min).	From 1.02 to 4.84	[72]
Neosepta AMX (Astom Corp.)	Immersion/ Polydopamine (PDA)	N.A.	Improved anti-organic fouling (transition time increased from less than 25 min to ~300 min) and anti-biofouling properties. Enhanced hydrophilicity (contact angle decreased from 70 θ to 45 θ).	From 2.5 to 5.0	[58,73] *
Neosepta AM-1, AM-2 and AM-3 (Astom Corp.)	Immersion/Sodium naphthalene sulfate and formaldehyde or polystyrene sulphonic acid	From 1.25 to 3.33, approximately	Higher ion exchange capacity.	N.A.	[56]
Neosepta ASE (Astom Corp.)	Co-deposition by immersion/ Mixed solution of polydopamine (PDA) and poly(sodium 4-styrene sulfonate) (PSS)	N.A.	Excellent organic antifouling properties (transition time increased from 240 min to 1200 min). Improved hydrophilicity (contact angle decreased from 78 θ to 58 θ) and stability.	From ~3.6 to ~4.5	[54]
Neosepta AMX (Astom Corp.)	Dip coating/ Polydopamine (PDA)	From 0.8 to 4.5	Validation of a theoretical model to obtain the charge density of the negatively charged layer	From 1.15 to 2.85	[41]
Neosepta AMX (Astom Corp.)	Dip coating/ L-PDA and 4,4′-diamino-2,2′-biphenyldisulfonic acid (DBSA)	From 1.25 to 2.13	Enhancement of the organic fouling resistance. Electrical resistance due to fouling reduced from 1.14 Ω·cm^2^ to 0.01 Ω·cm^2^	From 1.49 to 3.62	[74]
Home-made AEM from copolymer membranes composed of chloromethylstyrene and divinylbenzene	Immersion and refluxing/ Polyethylene polyamines (PEPDA)	From 1.20 to 3.03	Membrane hydrophilicity improved.	From 1.80 to 5.6	[75]
Heterogeneous Ralex AM-PES (Mega a.s.)	Coating (direct contact)/ Poly(acrylic) acid (PAA)	Sulfate rejection increased by 35%	Improved hydrophilicity (water contact angle decreased from 96 θ to 66 θ)	From 5.0 to 5.4	[55] *
Heterogeneous Ralex AMH (Mega a.s.)	Coating (sequential diffusion)/ Polyaniline (PANi) and perfluorocarbon cation-exchanger MF4-SK/PANi	N.A.	Increased ion exchange capacity, electrical conductivity and limiting current density. High mechanical and chemical stability.	N.A.	[76]
Neosepta AMX (Astom Corp.)	Adsorption/ Poly(ethyleneimine) (PEI)	Selectivity coefficients for SO_4_^2−^/Cl^−^, NO_3_−/Cl^−^, and SO_4_^2−^/NO_3_^−^ are reduced from 0.11 to 0.04, 0.71 to 0.24, and 0.21 to 0.08, respectively	The modified membrane became more selective towards monovalent anions	N.A.	[77]
AEM** (Ionics)	Coating by adsorption/ Olygourethane surfactants and Disodium salt α,ω-oligooxipropylene-bis(o-urethane-2.4,2.6-tolueneurylbenzene sulphonic acid)	N.A.	Power consumption reduced 1.7 times. Excellent anti-organic fouling properties	From 2.5 to 5.7	[57]
CJMA-2 (Hefei Chemjoy Polymer Material Co., Ltd., Hefei, China)	Layer-by-layer (LbL) deposition (7.5 bilayers)/ Poly(styrene sulfonate) and poly(ethyleneimine) (PEI)	From 1.10 to 2.44	Anti-organic fouling potential transition time improved by 38.4%. Enhanced hydrophilicity (contact angle decreased from 82.47 θ to 68.63 θ), and gross power density increased by 10% compared to Neosepta ACS.	From 2.8 to 3.3	[37] *
AEM Type I (Fujifilm Corp.)	Coating by LbL/ Poly(4-styrene sulfonate) and protonated poly(allylamine)	From 1.3 to 7.4	Increased Cl^−^/SO_4_^2−^ permselectivity in Diffusion dialysis	N.A.	[43]
TWEDA1 (Tianwei Membrane Technology Co.)	Coating via LbL (10.5 layers)/ Poly (sodium-p-styrene sulfonate), Poly (diallyldimethyl ammonium chloride) (PDDA), and graphene	From 1 to 11.5 (better performance than Neosepta ACS)	Improved separation efficiency of monovalent ions. Controlled water migration	From 1.81 to 2.31	[46]
Heterogeneous AEM ** (Zhe-jiang Qianqiu Environmental Protection & Water Treatment Co. Ltd.)	LbL deposition (10 layers max.)/ Glutaraldehyde (GA) and poly(ethyleneimine) (PEI)	From 0.42 to 0.55	Increased hydrophilicity (water contact angle decreased from 102.3 θ to 73.2 θ) and improved surface homogeneity	From 4.47 to 4.81	[78]
Neosepta AMX (Astom Corp.)	LbL deposition/Poly(sodium 4-styrene sulfonate) (PSS) and poly(allylamine hydrochloride) (PAH)	From 0.8 to 2.6	Improved antifouling properties (transition time increased from almost zero to ~150 min).	N.A.	[61]
AEM Type I (Fujifilm Corp.)	Electric-pulse LbL deposition (7.5 bilayers)/ Hydroxypropyltrimethyl ammonium chloride chitosan (HACC) and N-O-sulfonic acid benzyl chitosan (NSBC)	From 0.81 to 47.04 (higher value than those associated with Neosepta ACS and Selemion ASV)	Separation efficiency rising from –8.93% to 94.43%	From 1.31 to ~3.53	[45]
AEM Type I (Fujifilm Corp.)	Alternating current LbL assembly/ Poly(4-styrenesulphonic acid-*co*-maleic acid) sodium salt, 2-hydroxypropyltrimethyl ammonium chloride chitosan, and 1,4-bis(2′,3′-epoxypropyl) perfluoro-1-butane	From 0.81 to 4.87	Improved separation efficiency (from −8% to 62%). Improved antifouling characteristics against three foulants. The modified layer was stable after 96 h of operation.	N.A.	[44]
AEM Type I (Fujifilm Corp.)	Deposition/ Polydopamine (PDA) and sandwich alternating bilayers of poly(sodium 4-styrene sulfonate) (PSS)/hydroxypropyl trimethyl ammonium chloride chitosan-nano silver particles (HACC-Ag Np)	From 0.98 to 5.1	Higher anti-organic fouling potential (transition time enhanced from 60 min to 125 min). Improved hydrophilicity (contact angle decreased from 101.8 θ to 95.5 θ)	From 1.70 to 3.93	[79]
JAM-II-07 (Yanrun Co.)	Coating by Deposition/ Sulfonated reduced graphene oxide (S-rGO) nanosheets	From 0.72 to 2.30	Separation efficiency increased from −0.07 to 0.28	From 3.06 to 3.72	[80]
AEM ** (Fujifilm Corp.)	Electrodeposition/ Polydopamine (PDA) and N-O-sulfonic acid benzyl chitosan (NSBC)	From 0.78 to 2.20	Enhanced anti-organic fouling properties	From 1.3 to 1.94	[81]
Neosepta AEM ***	Electrodeposition/ Poly(ethyleneimine) (PEI)	From 0.79 to 4.2	The modified layer was stable up to 70 h of operation.	From 4.63 to 6.05	[35]
AEM ** (Fujifilm Corp.)	Alternate electrodeposition (9 bilayers)/ poly(sodium 4-styrene sulfonate) (PSS) and hydroxypropyltrimethyl ammonium chloride chitosan (HACC)	From 0.66 to 2.90	Separation efficiency improved from −0.19 to 0.28	From 1.31 to 4.52	[42]
Neosepta AMX (Astom Corp.)	Fouling deposition/ Sulfonated poly(2,6-dimethyl-1,4-phenylene oxide) (SPPO)	From 1.95 to 52.44, higher value than the one associated with Neosepta ACS	N.A.	From 2.4 to 2.83	[65]

Notations: $P_{\mathrm{SO}4^{2-}}^{{\mathrm{Cl}}^{-}}$ represents the membrane permselectivity between Cl^−^ and SO_4_^2−^ anions, respectively.* RED—related articles; ** no specific membrane name is reported; *** reported to be purchased from Fujifilm. If not specifically indicated the AEMs used are homogeneous; N.A.—not available.
